# Oxidative Stress and Nano-Toxicity Induced by TiO_2_ and ZnO on WAG Cell Line

**DOI:** 10.1371/journal.pone.0127493

**Published:** 2015-05-26

**Authors:** Akhilesh Dubey, Mukunda Goswami, Kamalendra Yadav, Dharmendra Chaudhary

**Affiliations:** 1 National Bureau of Fish Genetic Resources, Lucknow, Uttar Pradesh, India; 2 National Agri-Food Biotechnology Institute, Mohali, Punjab, India; Zhejiang University, CHINA

## Abstract

Metallic nanoparticles are widely used in cosmetics, food products and textile industry. These particles are known to cause respiratory toxicity and epithelial inflammation. They are eventually released to aquatic environment necessitating toxicity studies in cells from respiratory organs of aquatic organisms. Hence, we have developed and characterized a new cell line, WAG, from gill tissue of *Wallago attu* for toxicity assessment of TiO_2_ and ZnO nanoparticles. The efficacy of the cell line as an *in vitro* system for nanoparticles toxicity studies was established using electron microscopy, cytotoxicity assays, genotoxicity assays and oxidative stress biomarkers. Results obtained with MTT assay, neutral red uptake assay and lactate dehydrogenase assay showed acute toxicity to WAG cells with IC_50_ values of 25.29±0.12, 34.99±0.09 and 35.06±0.09 mg/l for TiO_2_ and 5.716±0.1, 3.160±0.1 and 5.57±0.12 mg/l for ZnO treatment respectively. The physicochemical properties and size distribution of nanoparticles were characterized using electron microscopy with integrated energy dispersive X-ray spectroscopy and Zetasizer. Dose dependent increase in DNA damage, lipid peroxidation and protein carbonylation along with a significant decrease in activity of Superoxide Dismutase, Catalase, total Glutathione levels and total antioxidant capacity with increasing concentration of exposed nanoparticles indicated that the cells were under oxidative stress. The study established WAG cell line as an *in vitro* system to study toxicity mechanisms of nanoparticles on aquatic organisms.

## Introduction

Nanoparticles have unique physicochemical properties due to their small size, large surface area and high reactivity. These special properties render them suitable for numerous applications such as therapeutics [1], environmental remediation [2], antimicrobial agents [3], transfection vectors [4], consumer products [5] and fluorescent labels [6]. Hence, nanotechnology based industries are growing rapidly leading to large scale production of engineered nanoparticles. Titanium dioxide (TiO_2_) and Zinc oxide (ZnO) are two such metallic nanoparticles that have been widely used in domestic and cosmetic products [7–8] and waste water treatment [9]. These nanoparticles are ultimately released to aquatic environments *via* bathing and sewage effluents [10–12] leading to increased chances of nanoparticle exposure to human and ecosystems. Studies with TiO_2_ particles have demonstrated respiratory toxicity and epithelial inflammation of the lung in rodents [13–15]. Most of the literature on toxicity of these nanoparticles had come from mammalian studies on respiratory exposure, or from *in-vitro* assays with mammalian cells [11].

Water bodies act as the sink for disposal of all the toxicants which after bioaccumulation ultimately leads to human exposure [16]. Fish, the most diverse group of vertebrates are thus of special importance for evaluation of ecotoxicants [17]. The *in vitro* cell culture systems are the preferred approach towards identifying the toxicity mechanisms of nano-sized materials. These systems are now widely used to facilitate hazard ranking of nanoparticles (NPs). It has been recommended that testing of nano-toxicants should be based on scientific paradigms which allow the screening of multiple toxicants. Xia et al were the first to propose that oxidative stress was a valid test paradigm for assessment of NP toxicity [18]. NPs induced production of reactive oxygen species (ROS) which created a redox imbalance. This leads to the physiological effect which is known as oxidative stress. Indicators of oxidative stress include changes in activity of antioxidant enzymes, levels of antioxidant molecules, damaged DNA bases, protein oxidation products, and lipid peroxidation products which are used to elucidate the toxicity mechanism of pollutants. Understanding the toxicity mechanism of nanoparticles on fish will assist not only in evaluating its impact on the aquatic environment but also in knowing its effects on human health [19].

Gills, the unique structure involved in respiration and osmoregulation, are the primary target and uptake sites of water contaminants [20]. Permanent gill cell lines have been used as model systems for ecotoxicological studies due to their higher control of the assay conditions, higher reproducibility and reduced variability of responses due to unavoidable stress [21]. Hitherto, very few cell lines developed in India have been used for *in vitro* toxicity studies. Recently few studies have been taken up for toxicity studies of aquatic pollutants [22–25]. The present study was thus aimed to evaluate the toxicity of the metallic nanoparticles using a new gill cell line from *Wallago attu*.

## Methods

### Specimens

Healthy juveniles of *W*. *attu* (5–10 g) were collected from the Gomti river, Lucknow and were kept in clean 6X3X2 ft^3^ fibre reinforced plastic (FRP) tanks at the wet lab facility of National Bureau of Fish Genetic Resources (NBFGR), Lucknow. The juveniles were acclimatized in hygienically maintained freshwater with proper aeration and frequent water exchange for a minimum period of one month. They were fed twice a day @ 2% of their body weight.

### Ethics Statement

The study was conducted at National Bureau of Fish Genetic Resources (26°47'15"N 80°56'9"E) as a part of project “Establishment of a National Repository at NBFGR, Lucknow for Conservation and Characterization of Fish Cell Lines”. The work was approved by “Institutional Research Committee” and by “Institutional Animal Ethics Committee, National Bureau of Fish Genetic Resources”. The research work was carried out at the institute where no specific approval is required. Director, NBFGR issued the permit for carrying out the research and may be contacted for further permissions. It is also confirmed that the studies did not involve endangered or protected species.

### Establishment of Gill Cell Line

In order to establish novel cell line from gill tissue of *W*. *attu*, we used our previously standardized protocols with minor modifications [26]. One month old fingerling was starved in sterile, aerated water containing 1000 IU/ml penicillin (Gibco) and 1000 μg/ml streptomycin (Gibco) for 24 hours at room temperature. The fish was then anaesthetized in MS-222 (Sigma Aldrich) solution and surface sterilized with 70% alcohol. The gill tissue was taken out aseptically, washed thrice in phosphate buffered saline (Gibco) containing 500 IU/ml Penicillin and 500 μg/ml Streptomycin and 2.5 mg/ml Fungizone (Gibco), minced in 0.05% trypsin-EDTA (Gibco) and then seeded into 25 cm^2^ standard cell culture flasks (Nunc). The explants were maintained in L-15 medium supplemented with 20% fetal bovine serum (FBS, Gibco, US origin) at 28°C. The flasks were daily monitored for attachment of explants, cell proliferation and migration. Morphology of cells was regularly observed using an inverted phase contrast microscope (Olympus Optical Co. Ltd). Every 4–6 days, half of the medium was replaced with fresh medium until passaging. Upon attaining 90% confluency, cells were dislodged with trypsin-EDTA solution and were subcultured at a ratio of 1:2. After the first passage, cells were regularly passaged at an interval of 5–7 days. Concentration of FBS in L-15 medium was gradually decreased from initial 20% to 10% by 20th passage.

### Characterization of Gill Cell Line

Growth characteristics of the cells were studied at different temperatures and FBS concentrations at 20^th^ passage. Cells were seeded at a concentration of 1 × 10^5^ cells in 25 cm^2^ tissue culture flasks and incubated at 18, 20, 24, 28 and 32°C for 7 days. Cells from triplicate flasks at each temperature were trypsinized and counted using a haemocytometer for a period of one week. Cell growth in different concentrations of FBS (5, 10, 15 and 20%) was studied at 28°C using the same procedure described above.

### Molecular Authentication

Authentication of cell line was carried out by amplification and sequence analysis of 16S rRNA and mitochondrial cytochrome oxidase subunit I (COI) gene. Mitochondrial 16S ribosomal RNA gene was amplified using 16SAR (5'-CGCCTGTTTATCAAAAACAT-3') and 16SBR (5'- CCGGTCTGAACTCAGATCACGT -3') primers [27] whereas the COI gene was amplified using FISHF1- 5'-TCAACCAACCACAAAGACATTGGCAC-3' and FISH R1-5'-TAGACTTCTGGGTGGCCAAAGAATCA-3' [28] primers. The PCR products were visualized on 1.2% agarose gels and the most intense products were selected for sequencing.

### Chromosomal Analysis

Chromosome analysis of WAG cells were done by preparing metaphase plates from exponentially growing cells at 30^th^ to 40^th^ passage according to the method described by Alvarez et al. [29]. WAG cells were grown to 80% confluency in 75 cm^2^ tissue culture flasks after which the medium was replaced with 10 ml of fresh medium containing 0.1 ml of 1 μg ml^-1^ colcemid solution (Sigma-Aldrich) and incubated at 28°C for 2 h. Cells were harvested by centrifugation (700 g, 5 min) and suspended in a hypotonic solution of 0.5% KCl for 10 min. The swollen cells were fixed in methanol (Merck Millipore): acetic acid (Merck Millipore) (3:1). Slides were prepared following the conventional drop-splash technique [30]. The slides were stained with 5% Giemsa for 10 min, made permanent using DPX mountant (Sigma Aldrich) and the chromosomes were counted.

### Immunocytochemistry

Morphology of the WAG cells was examined through the expression of monoclonal antibodies directed against Vimentin (V6630-CLONE 9 Sigma) and Cytokeratin (C2931-Clone C-11 Sigma) at passages no. 42. WAG cells were grown to 90% confluency on round coverslips in 12 well tissue culture plates (Nunc). Cells were washed with PBS and fixed in 4% p-formaldhyde (PFA). The fixed cells were washed twice in PBS, permeabilized with 0.1% Triton X-100, blocked with PBS containing 5% sheep serum and then incubated for 40 minutes at 37°C. Block was removed and 100 μl of 1:40 anti-Vimentin clone V9 dilution, and a 1:200 anti-pan cytokeratin clone-11 dilution was added in duplicate wells and incubated for overnight at 4°C. The cells were washed with PBS and were incubated for 30 min with 100 μl of 1:300 dilution of FITC-labelled anti-mouse IgG. The cells were again washed in PBS, mounted with 50% glycerol in PBS and were observed under fluorescence microscope. Appropriate controls for autofluorescence and secondary antibodies were included.

### Transfection Efficiency

WAG cells were characterized for their transfection efficiency using pEGFP-C1 plasmid in LTX and Plus Reagents (Invitrogen) following manufacturer’s instruction at passage 30. WAG cells were grown to 70% confluency in 12-well plates, rinsed with PBS and supplemented with 500 μl of fresh L-15 medium without serum. The plasmid (200ng of pEGFP-C1) was dissolved in 100 μl of OptiMEM and then 0.5 μl of plus reagent was added. The mixture was incubated for 5 min at 28°C. 2 μl of lipofectamine LTX was added to the mixture and incubated for 30 min at 28°C. The mixture was then added dropwise on WAG cells in 12 well plates. The medium was replaced after 6 hrs. The green fluorescence signals were observed after 18 hrs under a fluorescence microscope (Olympus).

### Nanoparticle Characterization

TiO_2_ (Sigma-Aldrich, Product number: 700347) and ZnO (Sigma-Aldrich, Product number: 721077) nanoparticle dispersions were purchased from Sigma Aldrich, USA. Stock suspensions were prepared in milliQ water and were characterized using electron microscopy. The working solution of nanoparticles were prepared in L-15/ex medium, sonicated and 5 μL of this solution were drop cast on copper grids and subsequently air-dried at room temperature. Simlarly, 5 μL of nanoparticle obtained from the manufacturer in water was mixed (without sonication) and directly drop cast on copper grids. The drop-coated grids were analyzed by a high resolution electron microscopy (SEM and STEM) (FEI Quanta 450 for SEM and FEI Tecnai G2 Spirit TEM for STEM) operated at an accelerating voltage of 20–30 kV. The particle size distribution was determined by Zetasizer Ver. 7.01 (Malvern, Instrument Ltd. UK) which uses a dynamic light scattering technique.

### Cell Culture and Nanoparticles Treatment

WAG cells, grown to 70–80% confluency in six-well plate were treated with 50 mg/L of ZnO and TiO_2_ nanoparticles for 24 h. After exposure, the cells were rinsed with PBS, trypsinized, and cell pellet was harvested by centrifugation at 1200g for 10 minutes. The cell pellet was then rinsed with PBS followed by fixation in glutaraldehyde solution. After dehydration in ethanol, the cells were finally resuspended in absolute ethanol and 5 μL of this cell suspension was drop-cast on copper grid and dried in air. The grids were then analyzed by a high resolution electron microscopy (STEM) (FEI Quanta 450 for SEM and FEI Tecnai G2 Spirit TEM for STEM) operated at an accelerating voltage of 30 kV with integrated energy dispersive X-ray (EDX) spectroscopy (EDX).

### Measurement of Acute Cytotoxicity

Cytotoxic effect of TiO_2_ and ZnO nanoparticles on WAG cells was assessed by MTT, neutral red (NR) uptake and lactate dehydrogenase (LDH) assay. Briefly 1 X 10^4^ cells/well were seeded in 96 well plates and incubated at 28°C for 24 hour. 90% confluent cells were then exposed to different concentrations of TiO_2_ (200mg/l to 1.56mg/l) and ZnO (50mg/l to 0.39mg/l) nanoparticles. Following 24 hour exposure, the media was aspirated off and fresh L-15/ex solution was added to each well. Nanoparticles were prepared and used for cytotoxicity assays in L-15/ex solution instead of complete L-15 medium containing FBS to reduce the effects of metal binding to serum proteins [31].

10 μL of MTT solution (Sigma Aldrich) (5 mg/ml) was added in each well and the plates were incubated for 4 h at 28°C. Following incubation the MTT containing medium was aspirated off from the microtiter plate and the intracellular formazan crystals were extracted and solubilized in DMSO (Sigma Aldrich). The plates were gently shaken for 10 min and the absorbance was recorded with the help of ELISA reader (Tecan) at 570 nm.

NR uptake assay was performed by incubating the nanoparticle exposed cells with 100 μl of 33 μg/ml neutral red solution prepared in L-15/ex media at 28°C for 2 h. The cells were then washed with NR fixative solution (0.5% (v/v) formaldehyde (Sigma Aldrich) and 1% (w/v) CaCl_2_ (Sigma Aldrich) in milliQ water). 100 μl/well of NR extraction solution (1% (v/v), acetic acid (Sigma Aldrich) and 50% (v/v) ethanol (Merck Millipore) in deionized distilled water) was then added to solubilize the lysosomal neutral red. The plates were gently shaken for 10 minutes and the absorbance was recorded with the help of ELISA reader (Tecan) at 530 nm.

Total LDH assay was performed on nanoparticle exposed cells in 96 well plate system using Lactic Dehydrogenase based *In Vitro* Toxicology Assay Kit, (Sigma, Catalogue no. TOX7) following manufacturer’s instruction. Results were expressed as mean of at least three replicates ± standard error.

### Estimation of DNA Damage

DNA damage following 24 hour exposure of TiO_2_ and ZnO nanoparticles on WAG cells at different concentration (50, 25, 12.5 and 0 mg/l) was evaluated using Single cell gel electrophoresis assay and cytokinesis-blocked micronucleus assay [32].

#### Comet Assay

Nanoparticle treated cells were embedded in 0.5% low melting agarose (Sigma Aldrich) layer between 1.0% normal melting agarose (Sigma Aldrich) and 0.5% normal melting agarose. The cells were lysed with high salt and detergent concentrations (100 mM EDTA (Sigma Aldrich), 2.5 M NaCl (Sigma Aldrich), 10 mM tris base (Bio Rad), 1% Triton X-100 (Sigma Aldrich), adjusted to pH 10) for 1 h. DNA was allowed to unwind (1 mM EDTA, 10% DMSO, 300 mM NaOH (Sigma Aldrich), pH 13) for 20 min and then subjected to electrophoresis in the same solution as for unwinding (25 V, 300 mA) for 15 min. After electrophoresis, the alkalis in the gels were neutralized by rinsing the slides in a neutralization buffer (0.1 M Tris pH 7.5) for 5 min. The slides were treated with methanol for 10 minutes, stained with 45 μl of 20 μg/ml ethidium bromide solution and viewed under a fluorescent microscope (Olympus Optical Co. Ltd). 1000 cells were analyzed and % tail DNA was measured to evaluate the extent of DNA damage. Results were expressed as means of at least three replicates ± standard error.

#### Micronucleus assay

Nanoparticle treated cells in chamber slides (Eppendorf) were washed with PBS and L-15/ex media with serum and 1.5 μg/ml of cytochalasin B was added for 48 hours. The cells were then rinsed with PBS and fixed *in situ* with absolute methanol and washed thrice with 70% methanol and allowed to air dry for several minutes. Chamber was removed and slides were stained with acridine orange and observed using fluorescent microscope (Nikon). 1000 binucleate cells were used to calculate the percentage of micronucleus in binucleate cells to evaluate the extent of DNA damage. Results were expressed as means of at least three replicates ± standard error.

### Estimation of Reactive Oxygen Species (ROS)

The detection of superoxide anion formation by the reduction of nitroblue tetrazolium (NBT) was performed using method of Wang et al [33]. Briefly 1 X 10^4^ cells/well were seeded in 96 well plates and incubated at 28°C for 24 h. Cells were then exposed to different concentrations of TiO_2_ and ZnO (50, 25 and 12.5 mg/l) nanoparticles. Following 24 hour exposure, the media was aspirated off and plates were washed twice with PBS. 100 μl of 0.1% NBT (Fermentas) was added in culture medium. The plates were incubated for 1 hour at 28°C. The cells were fixed in absolute methanol, washed thrice with 70% methanol and allowed to dry for several minutes. The reduced formazen within the WAG cells were dissolved sequentially in 120 μl of 2 M KOH followed by 120 μl of DMSO and read at 620 nm using KOH/DMSO as a blank. Results were expressed as means of at least three replicates ± standard error.

### Estimation of Lipid Peroxidation

The measurement of 'Thiobarbituric Acid Reactive Substances' (TBARS) is a well-established method for screening and monitoring of lipid peroxidation. TBARS assay is a simple, reproducible tool for assaying MDA-TBA adduct formed by the reaction of MDA and TBA under high temperature (90–100°C) and acidic condition. MDA-TBA adduct formed in cells treated with TiO_2_ and ZnO nanoparticles at varying concentrations (50, 25 and 12.5 mg/l) for 24 h was measured colorimetrically at 540nm using commercially available kit (Cayman, Item No. 10009055) following manufacturer’s instruction. Briefly, 2 X 10^7^ nanoparticles treated cells were sonicated in PBS and the resulting cell lysates were used for the assay. Cell lysate (100 μl) was mixed with 100 μl of SDS (BioRad) solution and the reaction was initiated by adding 4 ml of the color reagent. The vials were boiled for 1 h and the reaction was stopped by incubating the vials in ice for 10 minutes. The reaction mixture was centrifuged for 10 min. at 1,600 X g at 4°C. The MDA-TBA adduct formed in the reaction was measured colorimetrically at 540 nm using a microplate reader (Biotek, Synergy Mx Monochromator-Based Multi-Mode Microplate Reader). Standard curve was plotted using corrected absorbance value of each standard as a function of MDA concentration and the values of MDA for each sample were calculated from the standard curve using the following formulae:

$$MDA\;\left(\mu M\right)=\frac{\left(Ab_{cs}-b\right)}{m}$$

Where, Ab_cs_ = corrected absorbance of samples

b = y intercept of standard curve

m = slope of standard curve

Results were expressed as means of at least three replicates ± standard error.

### Estimation of Protein Carbonyl Content (PCC)

The reaction between 2,4-dinitrophenylhydrazine (DNPH) is a well established method to detect and quantitate protein carbonyls. The reaction forms a Schiff base to produce the protein-hydrozone which can be analyzed spectrophotometrically at an absorbance of 370 nm. Protein carbonyl content of nanoparticle exposed cells was determined using commercially available kit (Cayman, Item No. 10005020) following manufacturer’s instruction. Briefly, nanoparticle exposed cells were sonicated in cold MES buffer (50 mM, pH 6.7, containing 1 mM EDTA) and centrifuged at 10,000 X g for 15 min at 4°C. The supernatant was collected and treated with streptomycin sulfate at a final concentration of 1% in the sample for 15 minutes at room temperature. After incubation, the mixture was centrifuged at 6,000 X g for 10 minutes at 4°C. 200 μl of supernatant was taken in two tubes and 800 μl of DNPH was added to sample tubes whereas 800 μl of 2.5 M HCl was added to the control tubes. Both tubes were incubated in dark for 1 hour with intermittent vortexing of samples every 15 minute during the incubation. 1ml of 20% TCA was added to the tubes, vortexed and incubated on ice for five minutes. The pellet obtained after centrifugation at 10,000X g for 10 minutes at 4°C was again treated similarly with 10% TCA. The pellet then obtained was resuspended in 1 ml of (1:1) Ethanol/Ethyl Acetate mixture. The pellets were mixed thoroughly and tubes were centrifuged 10,000 X g for 10 minutes at 4°C. Washing of pellet with Ethanol/Ethyl Acetate mixture was done thrice. After the final wash, protein pellets were resuspended in 500 μl of guanidine hydrochloride and centrifuged at 10,000 X g for 10 minutes at 4°C to remove any left-over debris. 220 μl of supernatant from sample and control tubes were taken in 96 well plate and absorbance was measured at 370 nm using a microplate reader (Biotek, Synergy Mx Monochromator-Based Multi-Mode Microplate Reader). Total Protein carbonyl content of nanoparticle treated cells was determined using the following equation:

$$PCC\;\left(\frac{nmol}{ml}\right)=\left(\frac{CA}{0.011}\right)\;*\;\left(\frac{500}{200}\right)$$

where CA = corrected absorbance calculated by subtracting the average absorbance of controls from the average absorbance of samples.

Results were expressed as means of at least three replicates ± standard error.

### Estimation of Superoxide Dismutase Activity

Superoxide dismutases (SODs) are metalloenzymes which catalyzes the dismutation of superoxide anion to molecular hydrogen and hydrogen peroxide. Tetrazolium salts are utilized for detection of superoxide radicals generated by xanthine oxidase and hypoxanthine. One unit of SOD is defined as the amount of enzyme needed to exhibit 50% dismutation of the superoxide radical. SOD activity of the nanoparticle treated cells was measured using commercially available kit (Cayman, Item no. 706002) following manufacturer’s instruction. Briefly, the nanoparticle exposed cells were sonicated in cold buffer (20 mM HEPES buffer, pH 7.2, containing 1 mM EGTA, 210 mM mannitol, and 70 mM sucrose) and centrifuged at 1,500 X g for 5 min at 4°C. 10 μl of supernatant and standards were added to 96 well plates and 200 μl of diluted Radical detector was added to it. The reaction was initiated by adding 20 μl of diluted Xanthine oxidase and mixed by gently shaking the plate for few seconds. The plates were incubated on shaker for 20 minutes at room temperature and the absorbance was measured at 450 nm using a microplate reader (Biotek, Synergy Mx Monochromator-Based Multi-Mode Microplate Reader). Standard curve was prepared using 10 μl of standards having final SOD activity ranging from 0 to 0.25 U/ml. SOD activity for each sample was then calculated from the standard curve:

$$SOD\;\left(\frac{U}{ml}\right)=\;\left(\frac{0.23}{0.01}\right)\;*\frac{\left(LR_{s}-b\right)}{m}$$

Where, LRs = Sample linearized rate

b = y intercept

m = slope

Results were expressed as means of at least three replicates ± standard error.

### Estimation of Catalase Activity

Catalase is an antioxidant enzyme which is involved in detoxification of hydrogen peroxide. It exhibits peroxidatic function in which low molecular weight alcohols serve as electron donors. The enzyme reacts with methanol at optimal concentration of H_2_O_2_ leading to production of formaldehyde which is measured colorimetrically with 4-amino-3-hydrazino-1,2,4-triazole (Purpald). Catalase activity of the nanoparticle treated cells was determined using commercially available kit (Cayman, Item no. 707002) following manufacturer’s instruction. Briefly, the nanoparticle exposed cells were sonicated in cold buffer (50 mM potassium phosphate, pH 7.0, containing 1 mM EDTA) and centrifuged at 10,000 X g for 15 min at 4°C. The supernatant (20 μl) was collected followed by the addition of 100 μl of assay buffer and 30 μl of methanol in each well of the 96-well plate. Reaction was initiated by adding 20 μl of hydrogen peroxide as a substrate and incubated on shaker for 20 min at room temperature. The reaction was stopped by adding 30 μl of potassium hydroxide. To this mixture, 30 μl of 4-amino-3-hydrazino-5-mercapto-1,2,4-triazole (purpald) was added as chromogen, and incubated for 10 min followed by the addition of 10 μl potassium periodate. The purple color formaldehyde product formed was measured colorimetrically at 540 nm using a microplate reader (Biotek, Synergy Mx Monochromator-Based Multi-Mode Microplate Reader). Standard Curve was prepared using 20 μl of standards having formaldehyde concentration ranging from 0 to 150 μl. Formaldehyde concentration of samples was then calculated from the standard curve using the following equation:

$$Formaldehyde\;\left(\mu M\right)=\left(\frac{0.17}{0.02}\right)\;*\frac{\left(Ab_{s}-b\right)}{m}$$

Where Ab_s_ = sample absorbance

b = y intercept

m = slope

Catalase activity was then calculated using the following equation:

$$CAT\;activity=\frac{\mu M\;of\;sample}{20\;min.}=nmol/min/ml$$

Results were expressed as means of at least three replicates ± standard error.

### Estimation of Total Glutathione Level

Glutathione (GSH) is a tripeptide which serves as a nucleophilic co-substrate to glutathione transferases in the detoxification of xenobiotics. It is an essential electron donor to glutathione peroxidases in the reduction of hydroperoxides. GSH is assayed using the enzymatic recycling method in which the sulfhydryl group of GSH reacts with 5-thio-2-nitrobenzoic acid (DTNB, Ellman’s reagent) and a yellow coloured 5-thio-2-nitrobenzoic acid (TNB) is produced. GSTNB (mixed disulfide of GSH and TNB) is concomitantly produced which is reduced by glutathione reductase and TNB is produced at a rate directly proportional to concentration of GSH in the sample. Measurement of this TNB at 405 nm gives an accurate estimation of GSH in the sample. The change in GSH level of nanoparticle exposed cells was determined using a commercially available kit (Cayman, Item no. 703002) following manufacturer’s instruction. Briefly, nanoparticle treated cells were homogenized in cold MES buffer (50 mM, pH 6.0, containing 1 mM EDTA). The homogenate was centrifuged at 10,000 rpm for 15 min at 4°C, and the supernatant was collected and deproteinated. The samples (50 μl) were transferred to 96- well plate, and 150 μl of freshly prepared assay cocktail was added. The plates were then incubated in dark and the absorbance was read after 25 min at 405 nm using a microplate reader (Biotek, Synergy Mx Monochromator-Based Multi-Mode Microplate Reader). Standard Curve was prepared using 50 μl of standards having total GSH equivalents ranging from 0 to 16 μM. Total GSH for each sample was then calculated from the standard curve:

$$Total\;GSH=2*\frac{\left(Ab_{s}-b\right)}{m}$$

Where Ab_s_ = sample absorbance

b = y intercept

m = slope

Results were expressed as means of at least three replicates ± standard error.

### Estimation of Total Antioxidant Capacity

The overall antioxidant capacity provides an estimate of all of its constituents including vitamins, proteins, lipids, glutathione, uric acid, *etc*. The assay is based on the inhibition of 2,2’-Azino-di-[3-ethylbenzthiazoline sulphonate] (ABTS) oxidation by metmyoglobin. Total antioxidant capacity of the nanoparticle treated cells was measured by reading the absorbance of oxidized ABTS using commercially available kit (Cayman, Item no. 709001) following manufacturer’s instruction. Briefly, the nanoparticle exposed cells were sonicated in cold buffer (5 mM potassium phosphate, pH 7.4, containing 0.9% NaCl and 0.1% glucose) and centrifuged at 10,000 X g for 15 min at 4°C. 10 μl of supernatant and standards were added to 96 well plates and 10 μl of metmyoglobin along with 150 μl of chromogen was added to it. The reaction was initiated by adding 40 μl of hydrogen peroxidase working solution quickly. The plates were incubated on shaker for 5 minutes at room temperature and the absorbance was measured at 750 nm using a microplate reader (Biotek, Synergy Mx Monochromator-Based Multi-Mode Microplate Reader). Standard Curve was prepared using 10 μl of Trolox standards having final trolox concentration ranging from 0 to 0.330 mM Trolox. Antioxidant concentration of each sample was then calculated from the standard curve:

$$Antioxidant\;\left(mM\right)=\frac{\left(Ab_{s}-b\right)}{m}$$

Where Ab_s_ = sample average absorbance

b = y intercept

m = slope

Results were expressed as means of at least three replicates ± standard error.

### Statistical Analysis

All the experiments were performed in three independent experiments (triplicates) with six replicates for each exposure concentration while carrying out cytotoxicity assays. Data was analyzed with Graph Pad Prism 5. The individual data points of the concentration–response cytotoxicity charts are presented as the arithmetic mean percent inhibition relative to the control ± standard error. Statistical analysis of data was carried out using one-way analysis of variance (P≤0.05), followed by Dunnett’s multiple comparison tests (p≤0.05) wherever applicable.

## Results

### Development and Characterization of WAG Cell Line

**Fig 1:**
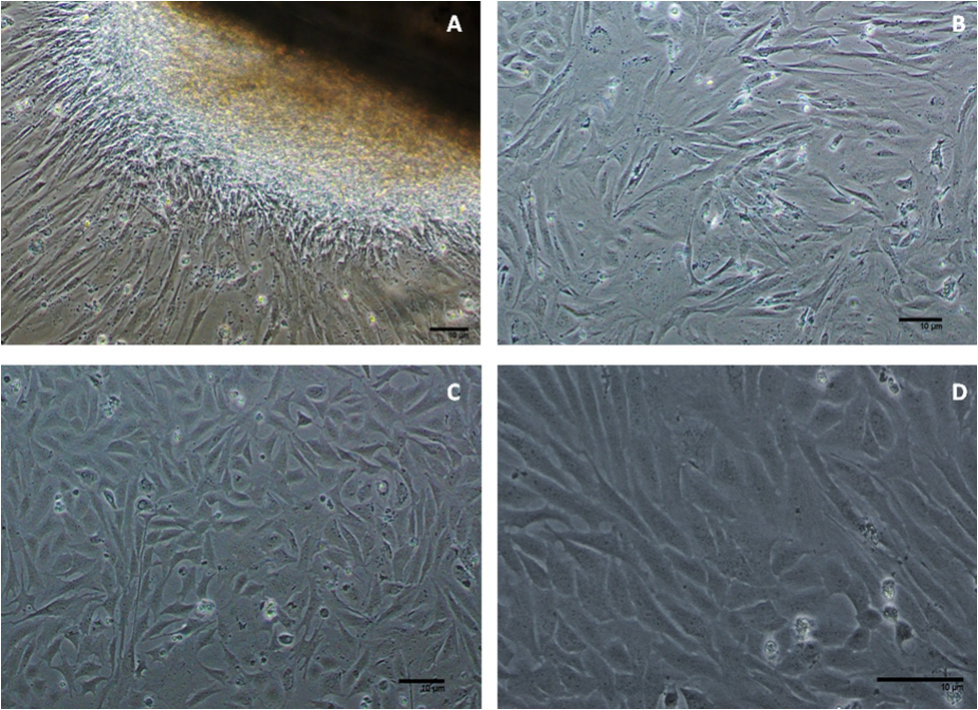
Photomicrograph of Gill cell line from *Wallago attu* (WAG); (A) Gill explant of *W*. *attu* (100X); (B) Cells of mixed morphology at paasage 5; (C) WAG cells at passage 40 (100X) (D) WAG cells at passage 40 (200X).

Primary cultures were initiated from trypsinized gill explants of *W*. *attu*. Cells emerged from both explants and trypsinized cell suspensions within 48 hours of addition of L-15 medium supplemented with 20% FBS at 28°C. Cells exhibited both epithelial and fibroblastic morphology and grew well to form monolayer during a time span of two weeks. Fibroblast like cells dominated in the culture at early passage. Decreasing FBS concentration @ 1% per passage to a final concentration of 10% FBS by 20^th^ passage (split ratio of 1:3) was efficient enough to maintain the culture giving a sub-culturing time of 3–5 days. To date, the cell line designated as WAG has been sub-cultured over 60 passage in a span of 1 year since its inception (Fig 1).

**Fig 2:**
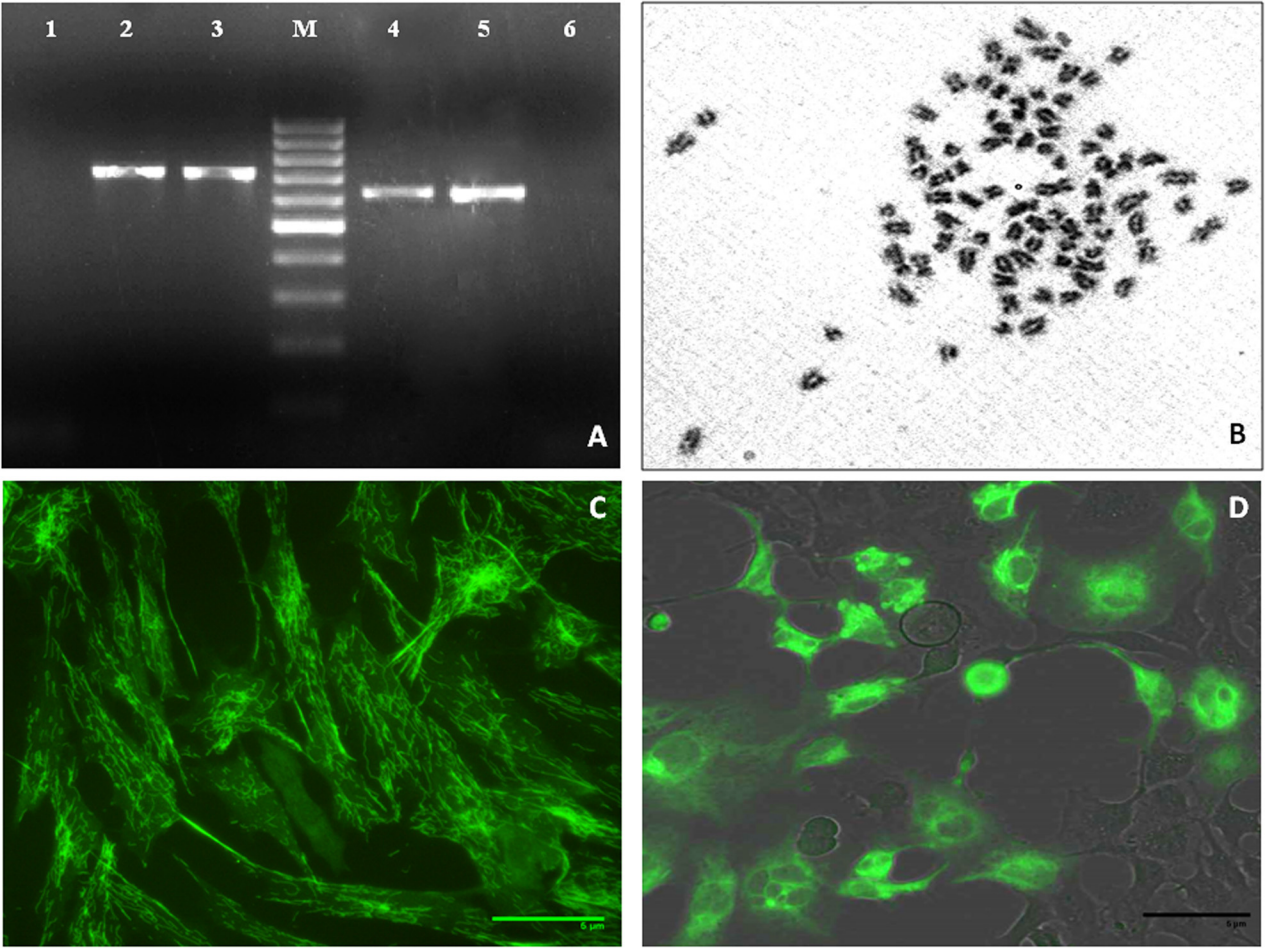
Characterization of WAG cell line; (A) amplification of 16S and COI DNA sequence (Lane 1and 6:PCR negative control; Lane 2: COI gene from WAG cells; Lane 3: COI gene from W. attu muscle tissue; Lane M: 100bp DNA ladder; Lane 4: 16S rRNA gene from WAG cells; Lane 5: 16S rRNA gene from *W*. *attu* muscle tissue); (B) chromosome spread of WAG cells at passage 40; (C) Morphological characterization of WAG fibroblastic cell line by Vimentin-FITC (400X); (D) Transfection of WAG cells using EGFP vector (400X).

Optimal temperature for WAG cell growth was observed at 28°C. Growth rate of WAG cells at 28°C increased with an increase in FBS concentration (5 to 20%). However, growth rate at 10% FBS was fairly good to reach confluency within 3 days at split ratio of 1:3. Cells origin was authenticated using gene amplification and sequence analysis of 16S and mitochondrial cytochrome oxidase I gene (COI) (Fig 2A). GenBank Accession No. for 16S rRNA and COI of WAG is KJ911222 and KJ911223 respectively. Chromosomal analysis of the cell line revealed diploid number ranging from 72 to 86 with a modal value of 86 chromosomes based on 56 metaphase plates (Fig 2B). Fibroblastic morphology of the cell line was confirmed using monoclonal antibody directed against Vimentin (Fig 2C). WAG cells were assessed for their application to gene expression and manipulation studies using transfection with pEGFP in WAG cells. After 24 h of transfection, 60% of WAG cells expressed green fluorescence of pEGFP plasmid (Fig 2D).

### Characterization of Nanoparticles

**Fig 3:**
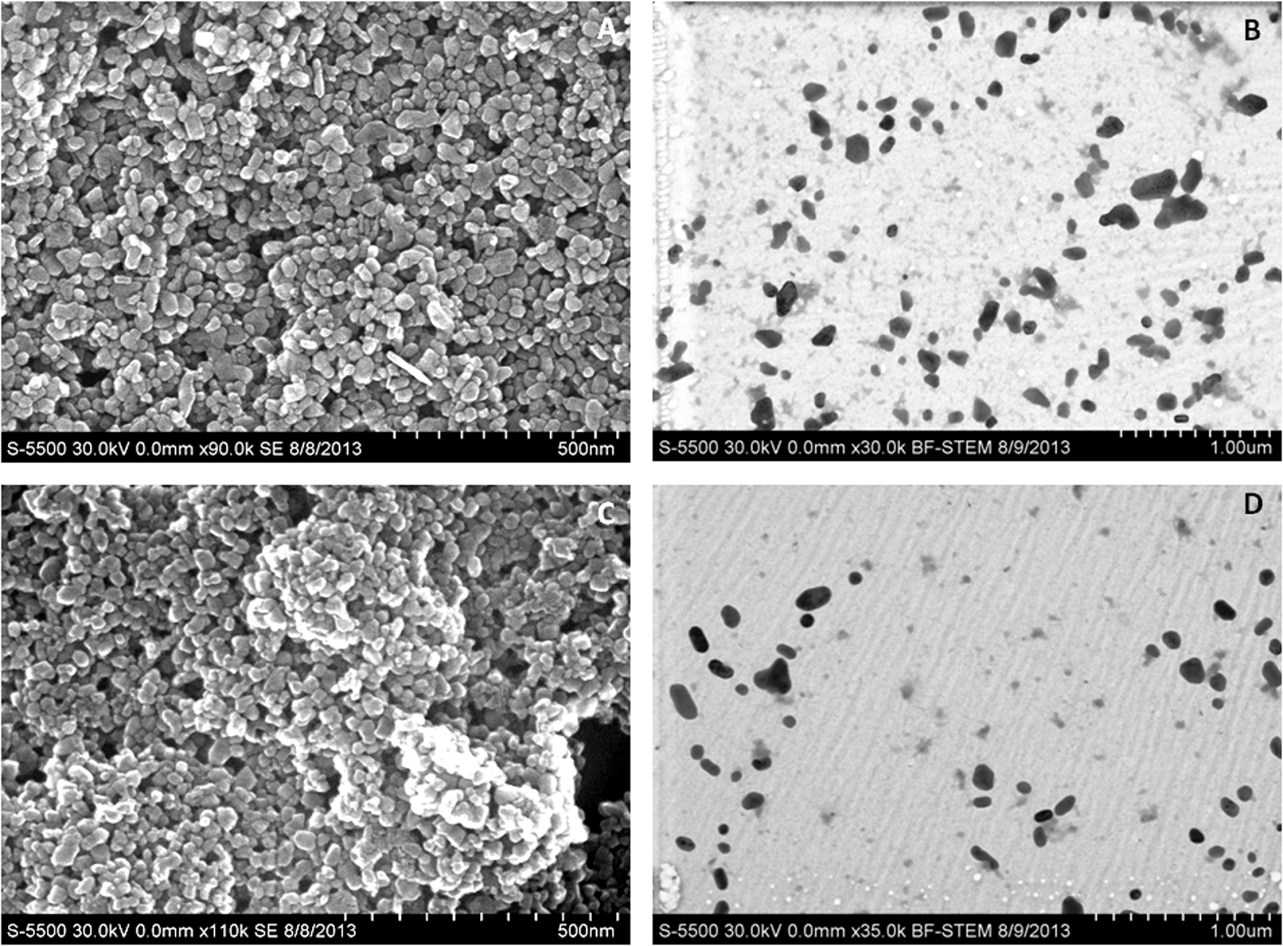
Electron micrograph of nanoparticles; (A) and (C) Scanning Electron image of TiO_2_ and ZnO respectively in water as provided by the manufacturer (with sonication); (B) and (D) Scanning Transmission Electron image of TiO_2_ and ZnO respectively, dispersed in L-15/ex medium through sonication.

Interaction of nanoparticles with surrounding cells or tissues has been attributed to their physicochemical properties. Nanoparticle dispersion and agglomeration have been reported to exhibit important role in their toxicity. Nanoparticles were characterized using electron microscopy (Fig 3 and Table 1). From the STEM images of nanoparticles after dispersion in L-15/ex solution it was observed that the nanoparticles were separated without considerable aggregation in the cells. The morphology of the nanoparticles present in media was similar to the nanoparticles obtained from the manufacturer (Secondary electron image of nanoparticles in water without dispersion). NP size as observed through TEM images for TiO_2_ (35.21 ± 14.1) and ZnO (25.12 ± 9.2) was in agreement with data provided by the supplier. However, the measured diameter of nanoparticles through zetasizer is 249.7 and 204.6 nm for TiO_2_ and ZnO nanoparticles respectively. This is in agreement to the fact that zetasizer overestimates the actual particle size for its measuring principle is based on hydrodynamic properties that are calculated by the Stokes-Einstein equation [34].

**Table 1:** Physical properties of investigated metallic nanopartciles.

S. No.	Nanoparticle	Source	Dia (nm)	Average Dia (nm) (Observed)	Agglomeration State	Measured Dia (nm) (Zaetasizer)
1	TiO_2_	Sigma Aldrich	<150	35.21±14.1	aggregate	249.7
2	ZnO	Sigma Aldrich	<100	25.12±9.2	aggregate	204.6

### Interaction of Nanoparticles with WAG Cells

**Fig 4:**
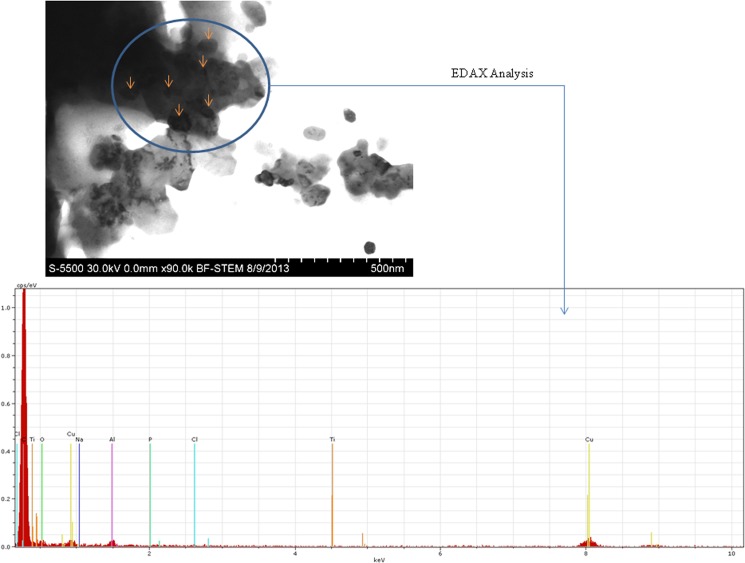
Cellular uptake of TiO_2_ nanoparticles is shown with the help of Scanning Transmission Electron image which was confirmed with EDAX analysis at several regions within the cell. Nanoparticles inside the cells are marked with the arrows. Carbon (C), Oxygen (O), Phosphorous (P), Sodium (Na), Chlorine (Cl) etc. are the peaks observed for cellular components with Ti peak representing the presence of uptaken nanoparticles. The Cu peak is because of the copper grid used in the experiment.

**Fig 5:**
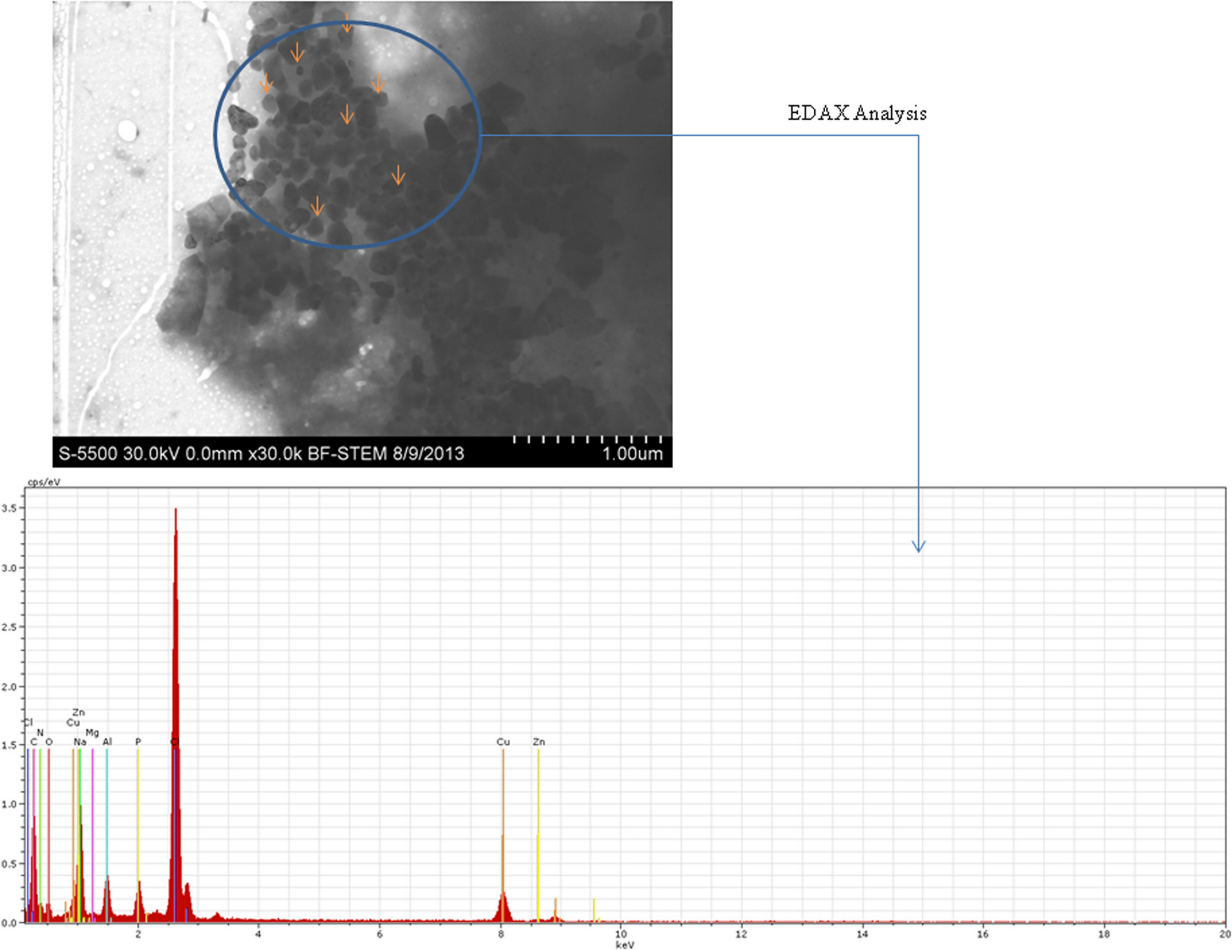
Cellular uptake of ZnO nanoparticles is shown with the help of Scanning Transmission Electron image which was confirmed with EDAX analysis at several regions within the cell. Nanoparticles inside the cells are marked with the arrows. Carbon (C), Oxygen (O), Nitrogen (N), Phosphorous (P), Sodium (Na), Chlorine (Cl) etc. are the peaks observed for cellular components with Zn peak representing the presence of uptaken nanoparticles. The Cu peak is because of the copper grid used in the experiment.

**Fig 6:**
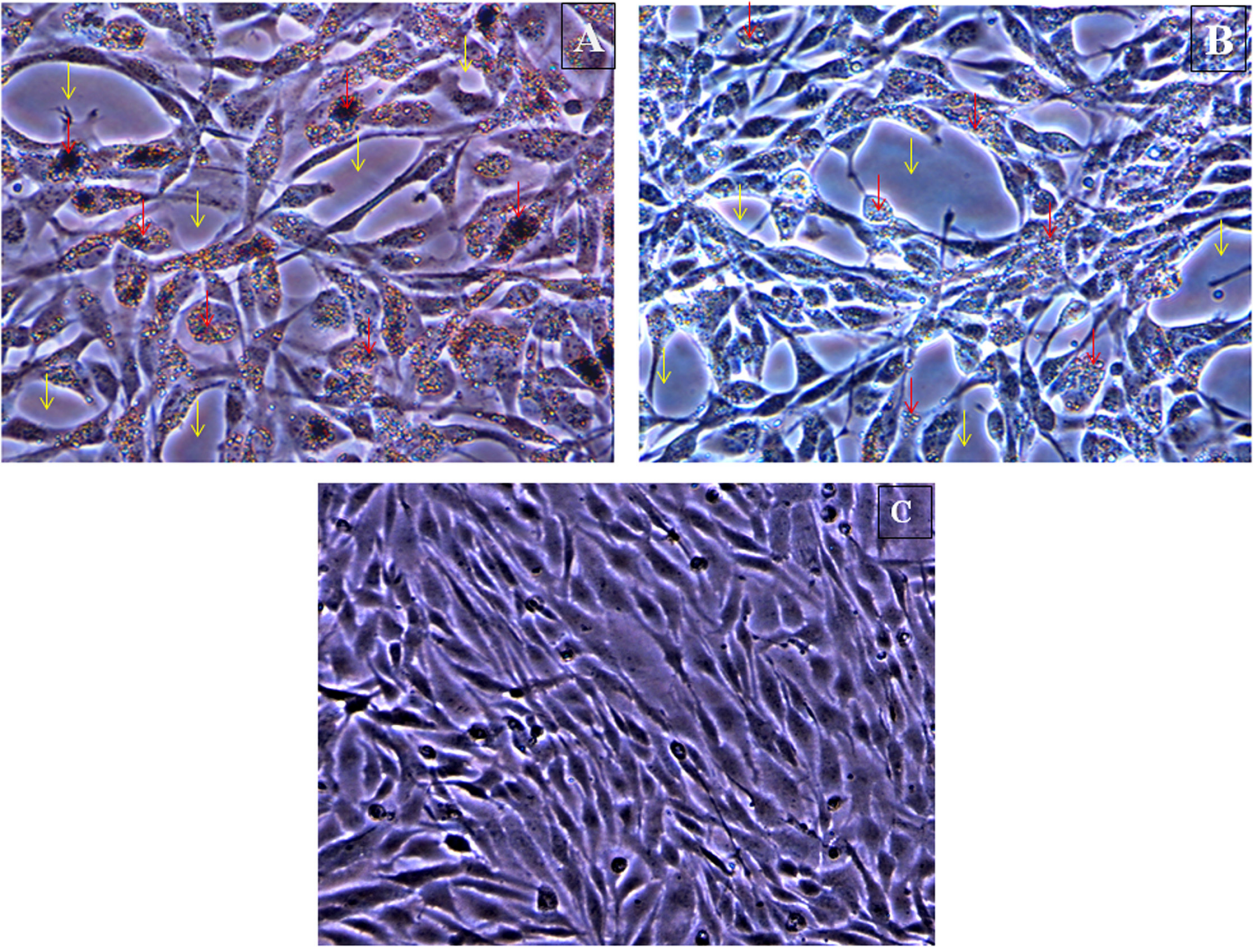
Interaction of nanoparticles with WAG cells A: TiO_2_; B: ZnO; C: Negative control. Nanoparticles inside the cells are shown with red arrows whereas the background area without cells is marked with yellow arrows. Complete absence of attached nanoparticles in background area confirms that the nanoparticles shown are inside the cells.

Images obtained from Scanning Transmission electron microscopy (STEM) revealed uptake of nanoparticles by treated WAG cells in L-15/ex medium (Figs 4 and 5). Presence of both TiO_2_ and ZnO nanoparticles inside the cells was also confirmed using Electron Dispersive X-ray (EDX) analysis (Figs 4 and 5). Photomicrographs obtained by phase contrast microscopy also complimented the results obtained by electron microscopy (Fig 6).

### Cytotoxicity of Nanoparticles to WAG Cell

**Fig 7:**
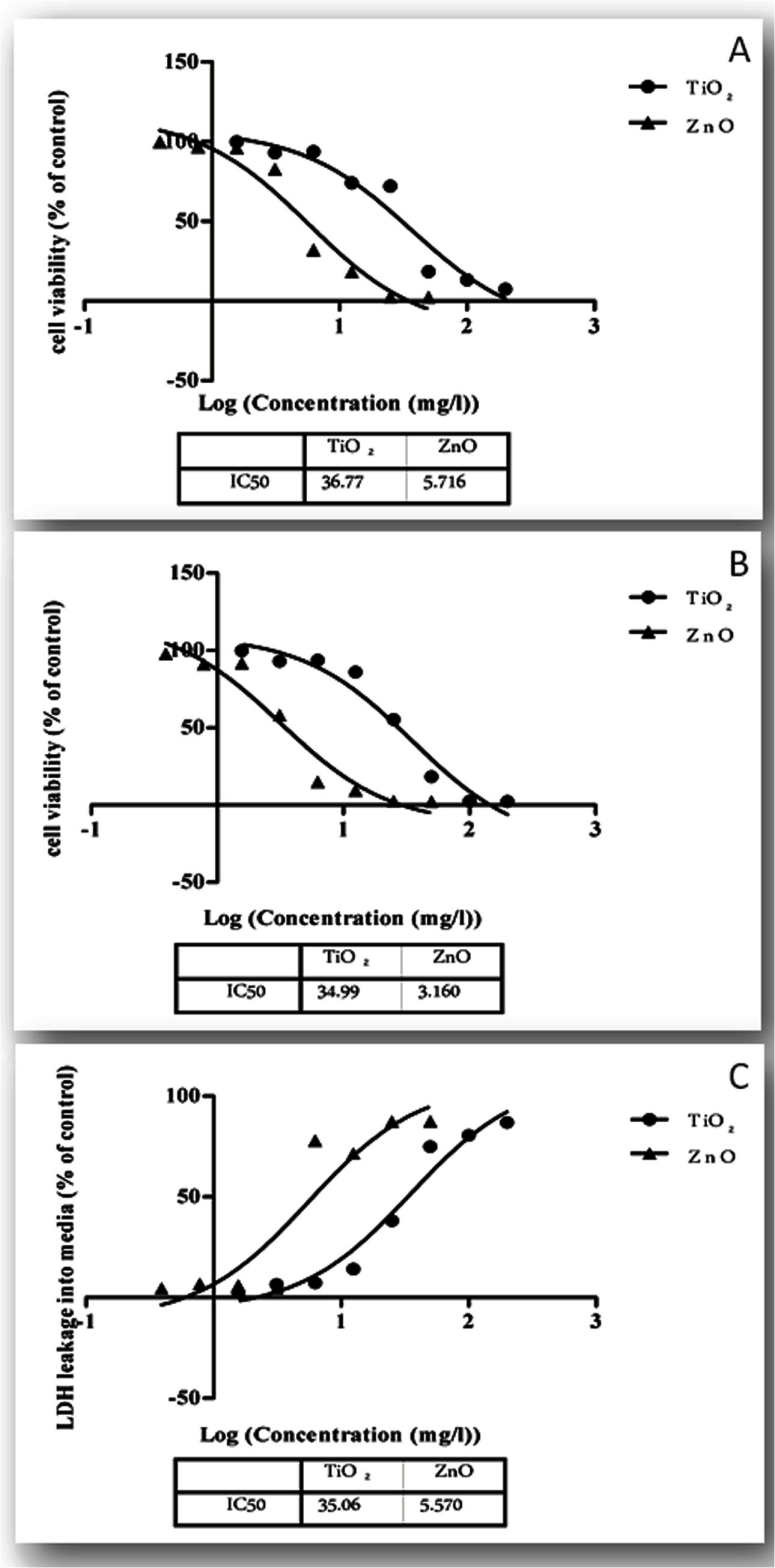
Drug dose response curves for the endpoint cytotoxicity assays upon exposure to nanoparticles A: MTT; B: NR; C: LDH assay.

Effect of TiO_2_ and ZnO nanoparticles following 24 h exposure to WAG cells was studied using MTT, NR and LDH assays. Both the nanoparticles exhibited significant dose dependent toxicity on WAG cells (Fig 7A, 7B and 7C). IC_50_ values (p≤0.05) calculated with MTT, NR and LDH assays were 25.29±0.12, 34.99±0.09 and 35.06±0.09 mg/l for TiO_2_ and 5.716±0.1, 3.160±0.1 and 5.57±0.12 mg/l for ZnO respectively (Table 2). Results revealed that ZnO nanoparticles were more toxic as compared to TiO_2_.

**Table 2:** Cytotoxicity effects two nano-sized metal oxides on WAG cells after 24 h incubation as quantified with the MTT, NR and LDH assay.

Nanoparticle	Exposure Period and endpoint	IC_50_(mg/L)	Hill slope	R^2^ value	95% Confidence Interval	
					IC_50_ (mg/L)	Hill slope
**TiO**_**2**_	24 hour MTT assay	25.29±0.12	-1.59±0.06	0.958	21.27 to 31.18	-2.01 to -1.16
	24 hour Neutral Red uptake assay	34.99±0.09	-1.41±0.13	0.963	30.27 to 40.18	-1.84 to -1.07
	24 hour LDH assay	35.06±0.09	1.51±0.23	0.945	31.05 to 19.34	1.97 to 1.04
**ZnO**	24 hour MTT assay	5.716±0.1	-1.44±0.08	0.986	5.31 to 6.12	-1.89 to -1.11
	24 hour Neutral Red uptake assay	3.160±0.1	-1.33±0.1	0.964	2.68 to 3.51	-1.74 to -0.97
	24 hour LDH assay	5.57±0.12	1.392±0.11	0.952	5.16 to 6.08	1.83 to 0.98

### Genotoxicity of Nanoparticles to WAG Cells

**Fig 8:**
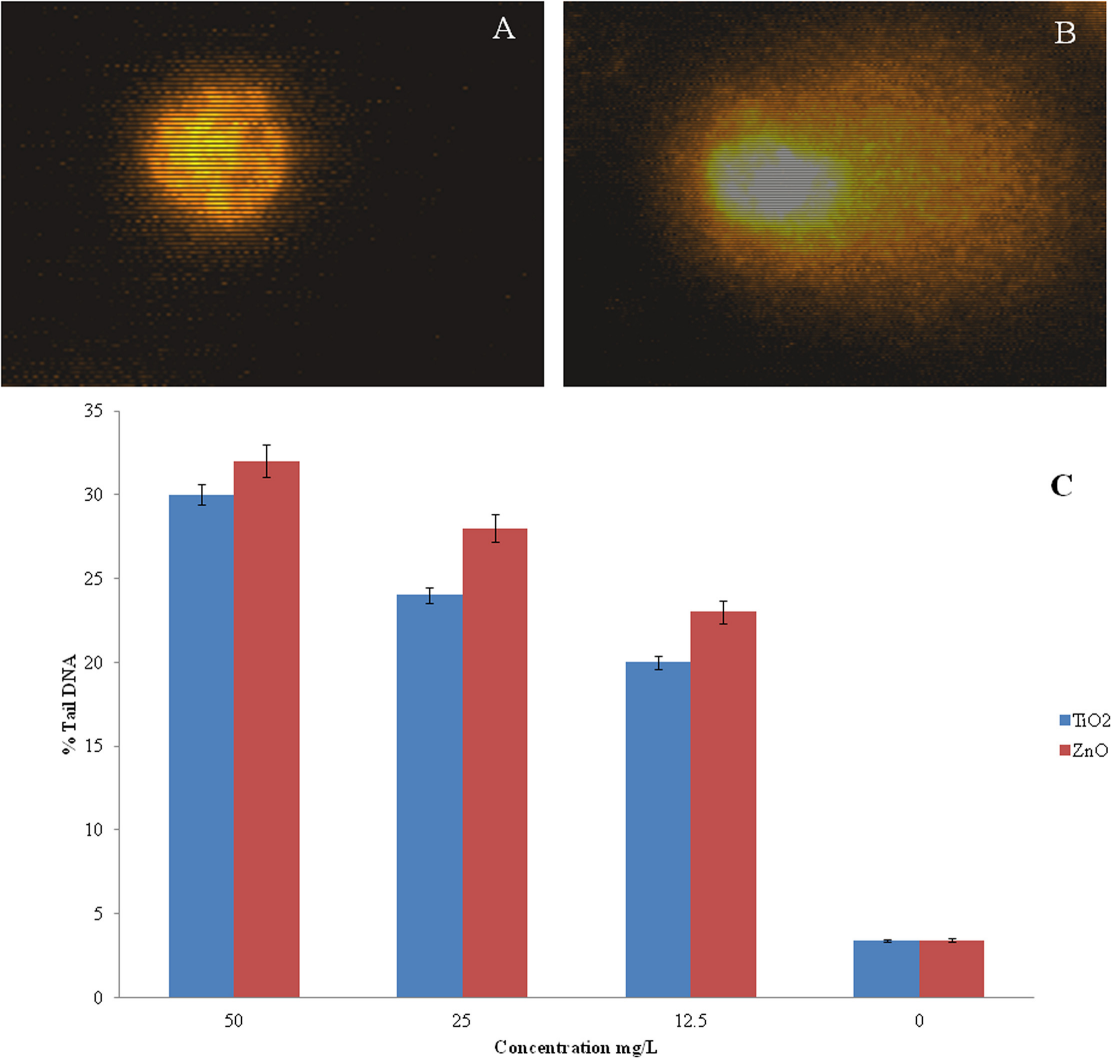
Comet assay analysis done for evaluating the DNA damage in cells upon exposure of nanoparticles; A: untreated WAG cell; B: nanoparticles treated WAG cell; C: % tail DNA observed in WAG cells after exposure to different concentration of nanoparticles.

**Fig 9:**
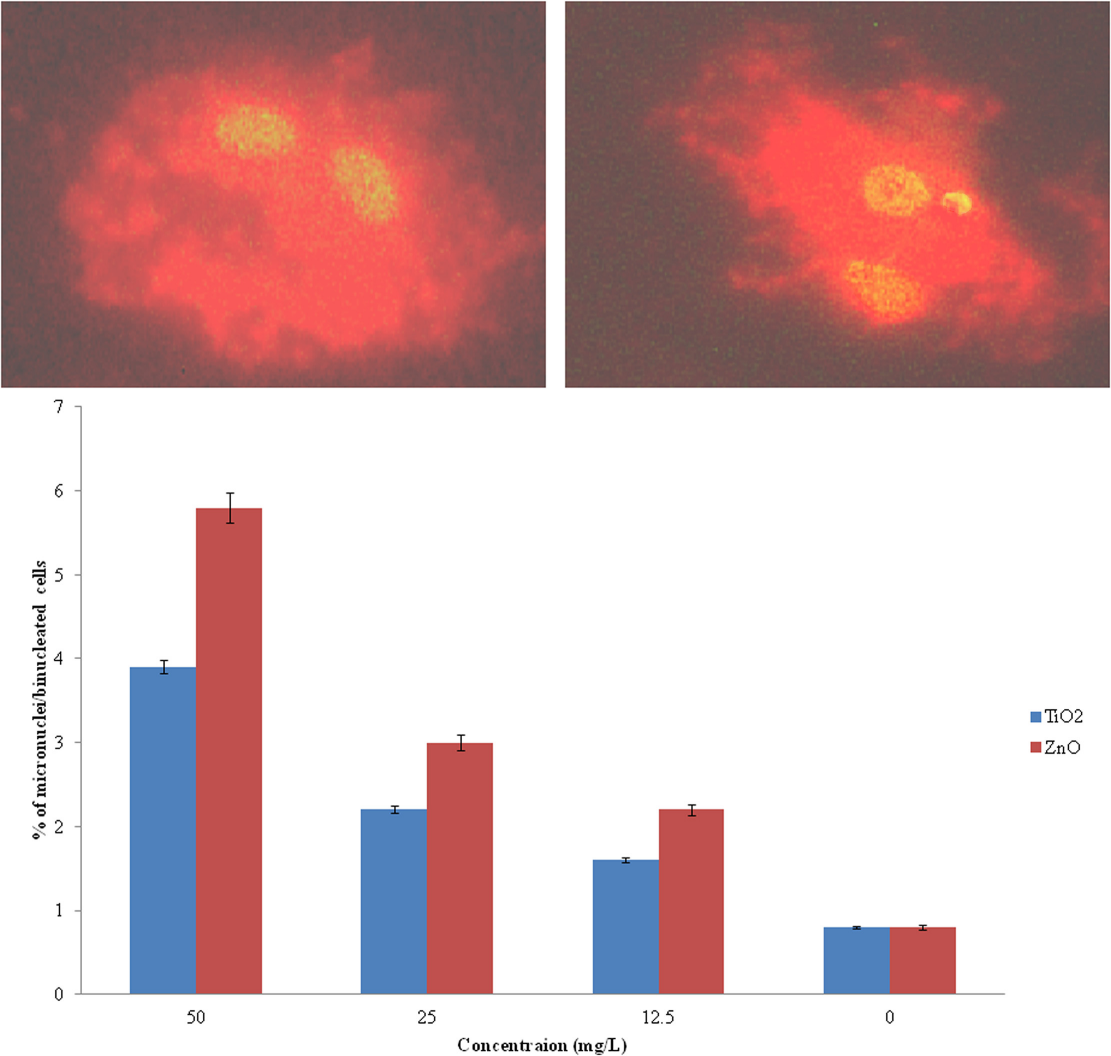
Micronucleus analysis done for evaluation of DNA damage in cells upon exposure to nanoparticles: A: untreated control WAG cell; B: treated WAG cell; C: % micronuclei in binucleated WAG cells after exposure to different concentration of nanoparticles.

Fig 8 shows a representative image of comet obtained after 24h exposure of nanoparticles on WAG cells. Dose dependent DNA damage was evident from % Tail DNA upon exposure to varying concentration of TiO_2_ and ZnO nanoparticles (Fig 8C). The results obtained from CBMNT assay further indicated the presence of chromosomal irregularities as compared to negative control cells (Fig 9).

### Assessment of Nanoparticle Induced Oxidative Stress on WAG Cells

**Fig 10:**
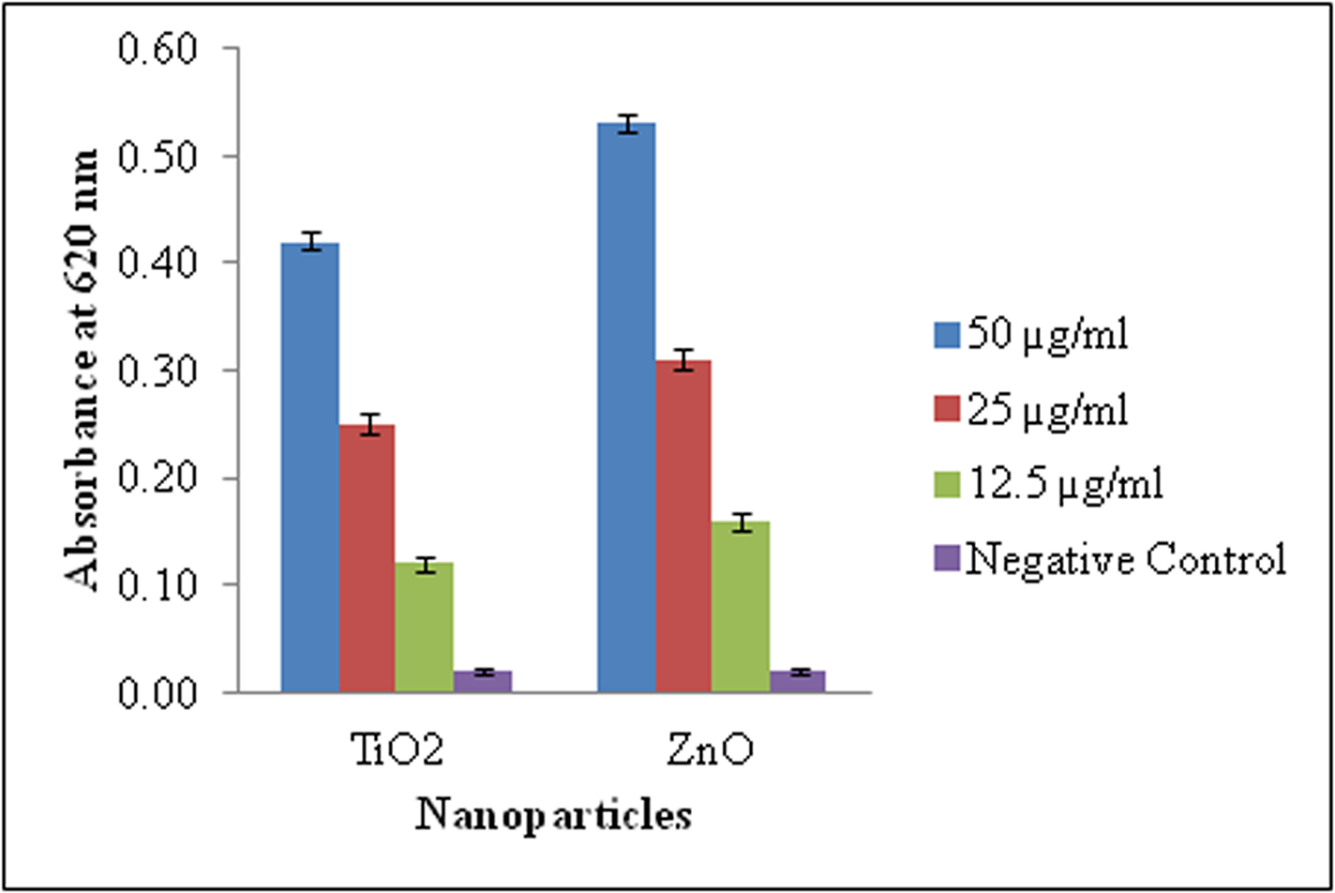
Detection of ROS (superoxide ion) by reduction of NBT in nanoparticle treated WAG cells.

**Fig 11:**
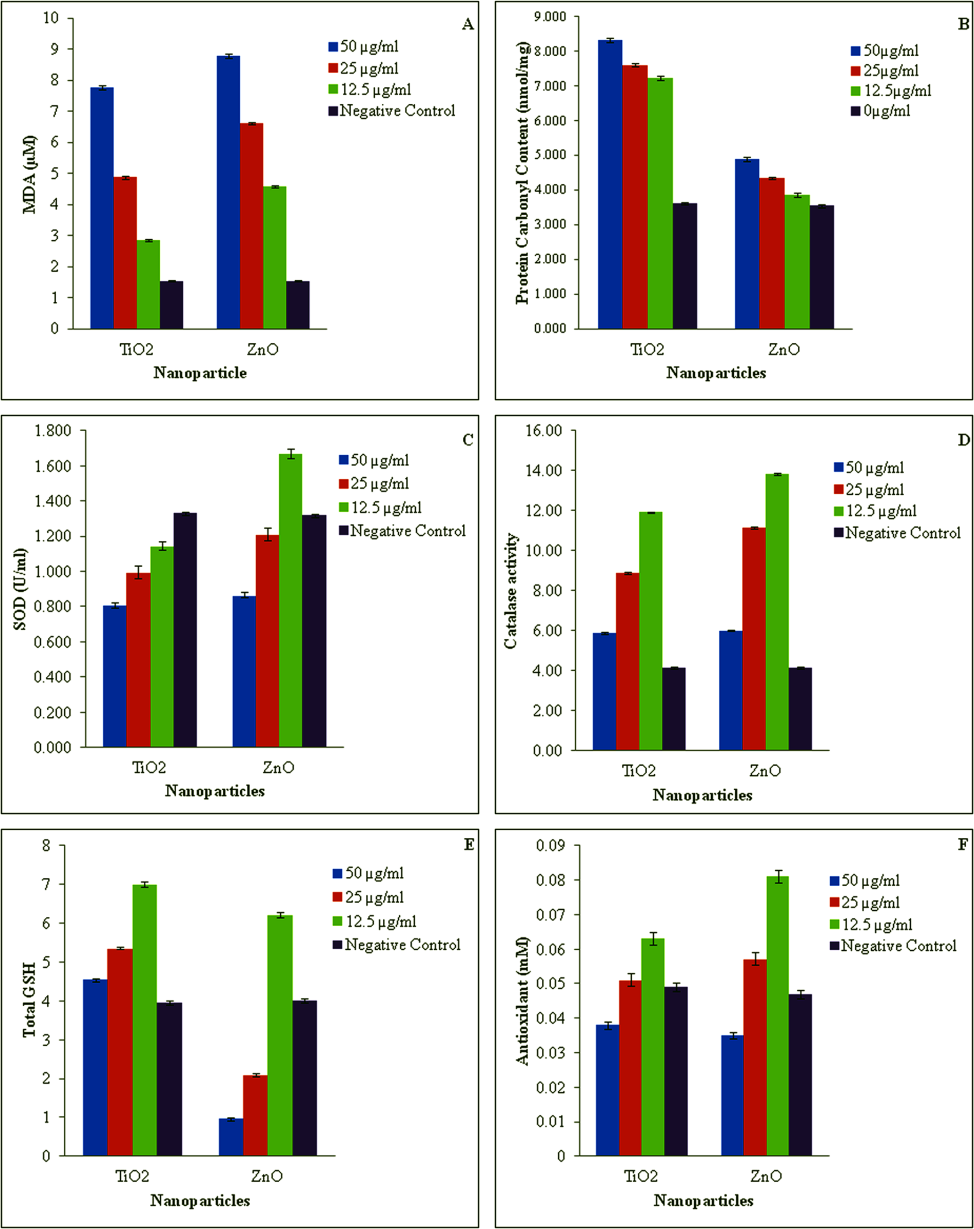
Evaluation of oxidative stress biomarkers against nanoparticles exposure to WAG cell line. A: MDA Concentration of nanoparticle treated WAG cells; B: Protein carbonyl content of nanoparticle treated WAG cells; C: SOD Activity of nanoparticle treated WAG cells; D: Catalase activity of nanoparticle treated WAG cells; E: Total Glutathione content of nanoparticle treated WAG cells; F: Total Antioxidant capacity of nanoparticle treated WAG cells.

TiO_2_ and ZnO nanoparticle treated cells exhibited dose-dependent increase in generation of ROS, lipid peroxidation and protein carbonyl content. The NBT assay measures superoxide-mediated production of formazan crystals which exhibited a linear increase in absorbance with increasing concentration of nanoparticles (Fig 10). Lipid peroxidation which reflects cellular membrane damage was evident as MDA levels were found to be significantly elevated at the three exposure concentration (12.5, 25 and 50 mg/l) of TiO_2_ (84.96%, 214.06% and 405.73%) and ZnO (198.18%, 330.28% and 471.81%) nanoparticles respectively (Fig 11A). Damage at protein level was revealed by 99.97, 110.45 and 130.45% increase in protein carbonyl content of TiO_2_ nanoparticle exposed WAG cells (Fig 11B). Similar results were obtained for ZnO nanoparticle treated WAG cells with 8.74, 22.33 and 37.86% increase in protein carbonyl content as compared to the negative control cells.

Dose dependent decrease in the activity of antioxidant enzymes i.e. SOD and Catalase was observed in nanoparticles treated WAG cells at test concentrations. Enzyme activity of SOD expressed as % of control decreased significantly to 86, 74 and 61% for TiO_2_ treated cells whereas a significant reduction to 126, 92 and 65% activity was observed for ZnO nanoparticle treated cells (Fig 11C). The other antioxidant enzyme i.e. Catalase, exhibited a similar response with a reduction to 188, 114 and 42% enzyme activity in TiO_2_ nanoparticle treated cells. For ZnO nanoparticle treated cells the enzyme activity reduced to 235, 169 and 44% of negative control cells (Fig 11D).

Total GSH (tGSH) is an important non-enzymatic component of cellular defense against oxidative stress. A change in tGSH level is generally thought to be an adaptive response of cells against oxidative stress. Dose-dependent depletion of tGSH was observed in nanoparticle exposed WAG cells. tGSH depleted to 176.36, 134.99 and 114.48% of control for TiO_2_ nanoparticle treated cells. Similarly, tGSH levels were depleted to 154.97, 51.98 and 23.94% in ZnO nanoparticle exposed WAG cells (Fig 11E).

Total antioxidant capacity of WAG cells upon exposure to varying concentrations of TiO_2_ and ZnO nanoparticles was assessed to demonstrate the status of oxidative stress induced by these nanoparticles (Fig 11F). Significant dose dependent decrease to 151, 86 and 29% of total antioxidant potential was observed in TiO_2_ exposed cells. Similar results were obtained for ZnO treated cells with 202, 128 and 72% of total antioxidant capacity as compared to negative control cells.

## Discussion

Metallic nanoparticles are able to cross the protective cellular defenses and disseminate into various organs of an aquatic organism. Gills are the exquisite organs which provide a thin entry route for these nanoparticles. Gill cell lines have been described as an ideal system for toxicity studies of aquatic pollutants [21]. Several reviews on use of fish cell lines for toxicology studies have been published [31, 35–36]. Among these, gill cell lines have been reported to be useful for studying toxicity of aquatic pollutants at bronchial level in greater detail as compared to *in vivo* studies. Nanoparticles have been described to have toxic effects on pulmonary targets [37–38]. Gill cell lines have been utilized for evaluation of toxicity caused by aquatic pollutants and good correlations with data observed from live fish studies have been reported [39]. Recently, significant correlation between *in vivo* and *in vitro* toxicity of silver nanoparticles to gill cell lines of *L*. *rohita* and *C*. *catla* has been reported [40]. Hence, in the present study we report establishment of a new gill cell line from freshwater shark, *W*. *attu* for toxicity studies of nanoparticles on respiratory cells of fish. The cell line is maintained at National Repository of Fish Cell lines (NRFC) established at National Bureau of Fish Genetic Resources, Lucknow and is available to researchers for further research. The established cell line was able to support growth at wide range of temperatures (24–32°C) in L-15 medium supplemented with 10% FBS. The cell line is stable with a model chromosome number of 86. The good transfection efficiency observed with the cell line also makes it useful for RNAi studies of genes involved in nanoparticle toxicity. Thus, it can be used as an ideal and cost effective system for toxicity studies without the need of specialized incubators. WAG, will not only be of great importance for assessing toxicity of nanoparticles in aquatic organisms but also for understanding the mechanism of action of these nanoparticles.

TiO_2_ and ZnO nanoparticles were selected for the study due to the consistent increase in synthesis and applications of these nano sized metallic oxides. Titanium is a harmless, non-toxic metal whereas zinc is essential for cells. TiO_2_ nanoparticles have been suggested to induce local vascular injury in bronchial capillary bed causing bronchial aneurisms and microvascular dysfunction in rats [41]. Physicochemical properties that are important for nanoparticle toxicity were analyzed using electron microscopy and were found to be in accordance with other studies [42–44]. Dynamic light scattering (DLS) was used for determining secondary size and extent of aggregation of these nanoparticles. The hydrodynamic diameter of nanoparticles in L-15/ex medium was higher as calculated by Zetasizer and the nanoparticles existed in unequal distribution of larger aggregates. *In vivo* studies of nanoparticle toxicity have concluded in nanoparticle induced sub-lethal toxicity involving respiratory distress, oxidative stress, organ pathologies and induction of antioxidant defense systems [45]. They have hypothesized that this sub-lethal effect of TiO_2_ nanoparticles is due to indirect systemic toxic effects caused by surface adhesion of nanoparticles rather than uptake into tissues. In our study, we have demonstrated the uptake and internalization of the nanoparticles with the help of optical and electron microscopy (Figs 3–6). The EDAX analysis of the cross section inside the cell shows a clear peak of Titanium and Zinc element (peaks of Ti and Zn) which establishes the uptake of nanoparticles by the cells. However many other peaks are also seen which demonstrate the elemental composition of the cell whereas the peak of Cu reflects the presence of the copper grid. Internalization of nanoparticles may cause metal catalyzed oxidation leading to these sub-lethal effects. This is in accordance with the study of metallic nanoparticles (TiO2, ZnO and CdS) on human kidney cells in which these nanoparticles are reported to be taken up by the cells without affecting their morphology. These nanoparticles are known to be aggregated in vesicles of IP15 and HK-2 cells [42, 46]. Handy et al. reviewed the possible mechanisms of ADME (absorption, distribution, metabolism and excretion) for nanoparticles in aquatic organisms [47]. Different mechanisms for uptake of nanoparticles have been proposed. Clathrin or caveoli mediated endocytosis of gold nanoparticles [48] and macro-pinocytosis have been reported as possible mechanism for active endocytosis of these nanoparticles [49]. Further, diffusion of gold, silica [50] and silver [51] nanoparticles through nuclear pore has been reported. Handy et al. has described the physiological mechanism of nanoparticles excretion in fish [47]. The molecular weight cutoff for excretion through glomeruler filter of kidney is 60 kDa or 8nm, hence fish utilize liver as the most likely route for excretion of metallic nanoparticles [47]. Recycling (exocytosis) of contents from vesicles has also been considered as a possible mechanism for excretion of metallic nanoparticles [52].

Biochemical endpoint assays viz. MTT, NR uptake and total LDH concentration were used to assess the cytotoxicity potential of these metallic nanoparticles. The results obtained with these colorimetric assays demonstrated a dose dependent decrease in cell viability. IC_50_ values following 24 hour treatment of nanoparticles was calculated to express the extent of toxicity caused by these nanoparticles. Comparison of IC_50_ values obtained by these assays indicated that ZnO is more toxic than TiO_2_ nanoparticles. This data is consistent with the results obtained from *in vivo* acute toxicity of these nanoparticles on zebra fish in which 96 hour LC_50_ values of 124.5 mg/l and 4.92 mg/l was reported for TiO_2_ and ZnO respectively [34]. However, the 24 hour IC_50_ value for TiO_2_ and ZnO nanoparticles in human skin fibroblast was found to be 2696±667 ppm and 49.56±12.89 ppm respectively [53]. The higher sensitivity of the nanoparticles to aquatic organisms may be attributed to the presence of higher content of unsaturated fatty acids in the cell membrane as these polyunsaturated fatty acids are more prone to oxidative damage [54]. Cytotoxicity of these nanoparticles has been compared to their bulk size particles on the basis of size, degree of aggregation, chemical composition, solubility, oxidation status and cell type [42]. In the current study, the average particle size and aggregate size of ZnO nanoparticle was smaller than TiO_2_ which reflects the observed toxicity of ZnO particle being higher than TiO_2_. Notter et al. reviewed toxicity of nanoparticles of different sizes and concluded that it isn’t the dominant factor which determines nanoparticle toxicity [55]. They didn’t find any correlation between particle size and toxicity of nanoparticles. However, studies with similar agglomerated size of nanoparticles had also differed in their cytotoxicity potential [56]. Toxicity of nanoparticles has thus been considered as a complex and multidimensional process in which particle size is one important parameter for nanoparticles characterization [55]. Cytotoxicity observed with these nanoparticles has also been attributed to their solubility [57].

Dose dependent increase in DNA damage was observed in nanoparticle treated WAG cells. Appearance of comet like tail indicates presence of DNA strand breaks (repairable damage to DNA) upon electrophoresis. Percent of DNA observed in the comet tail measures the extent of DNA damage. ZnO nanoparticles were again found to be more genotoxic as compared to TiO_2_ nanoparticles. This result was further complemented by the presence of micronucleus (irreparable DNA damage) in cytokinesis blocked nanoparticle treated cells. Reactive oxygen species has been attributed to cause gentotoxicity in cells exposed to metallic oxide nanoparticles [58–59]. Accumulation of these nanoparticles has been observed around nucleus (Fig 6). Due to the small size of nanoparticles few of them may diffuse into the nucleus through nuclear pore from where protein transport takes place. These nanoparticles can further augment the DNA damage caused by ROS.

In order to understand the toxic mechanism of nanoparticle, we studied several markers of oxidative stress including lipid peroxidation, protein carbonyl content, ROS production, total Glutathione concentration, SOD activity, Catalase activity and total antioxidant potential. Oxidative stress has been well documented as a common mechanism for nanoparticle induced cell damage [57]. Study of silver nanoparticle on BRL 3A rat liver cell line [60], human lung fibroblast cells (IMR-90) and human glioblastoma cells (U251) [51] have reported disturbences in oxidative stress markers and influence on respiratory chain. Many metallic nanoparticles have been studied and were reported to exert their toxicity through oxidative stress. Metallic nanoparticles in the study may liberate free metal ions in the cytoplasm upon surface oxidation. A similar mechanism for Silver [51], cobalt and nickel [61] has been reported. In the current study, ROS production was measured using NBT reduction which measures superoxide mediated production of formazan crystals. A dose dependent increase in ROS production was observed which may be due to higher surface reactivity of these nanoparticles or due to increased solubility of the metallic counterparts [34]. The increased production of ROS can oxidize cellular macromolecules. Both the nanoparticles caused an increase in lipid peroxidation and protein oxidation in a dose dependent manner. The results are in accordance with the data obtained from *in vivo* assays in zebra fish [34] and rainbrow trout [45] in which an increase in lipid peroxidation has been demonstrated in gill cells following exposure to these metallic nanoparticles. It has been proposed that TiO_2_ nanoparticles in direct contact with gill cells generate ROS in presence of light [45]. In our study we have observed internalization of nanoparticles which may further contribute in elevation of intracellular ROS production leading to increased level of lipid peroxidation. Poly unsaturated fatty acids (PUFAs) are of utmost importance to aquatic organism as it helps in maintaining membrane fluidity at lower temperature [54]. These PUFAs are more prone to oxidation and thus higher degree of peroxidation is observed in aquatic organisms as compared to mammalian cells [54]. Protein oxidation in form of protein carbonyl contents are produced in two ways. Oxidation of protein predominantly occurs via metal catalyzed oxidation and secondarily via oxidized macromolecules produced by ROS which could be a possible explanation of high protein damage observed in the nanoparticles treated cells [54]. Level of non-enzymatic antioxidant (tGSH) and activity of antioxidant enzyme SOD and catalase have always been considered as important biomarkers to study antioxidant defense system in animals. Dose dependent depletion of tGSH and decrease in activity of antioxidant enzymes has been observed. At lower dose of (25 and 12.5 mg/l) TiO_2_ and ZnO nanoparticle an increase in activity of antioxidant enzymes have been observed which can partly be explained by the fact that initial increase in ROS can lead to transcription of redox sensitive genes. Genes for SOD and CAT enzymes are two such genes whose transcription is increased in this manner [54]. Moreover, the antioxidant potential of the nanoparticle induced WAG cells was also found to decrease significantly. The activity of antioxidant enzymes and total antioxidant potential may increase due to mild oxidative stress which is associated with an increase in their synthesis. However, severe oxidative stress causes a decrease in the level of these antioxidant enzymes and hence in total antioxidant potential.

## Conclusion

In the present study, a new gill cell line was established and used as an *in vitro* model for toxicity studies of nanoparticles at bronchial level in aquatic organism. The IC_50_ values obtained with endpoint assays were found to be in concordance with values obtained previously through *in vivo* and *in vitro* toxicity experiments. Nanoparticle uptake by the cells and an increase in ROS production was observed which has resulted in an increased DNA damage, lipid peroxidation and protein carbonylation. Activity of enzymatic markers of oxidative stress i.e. Superoxide Dismutase (SOD) and Catalase along with non enzymatic components i.e. level of total glutathione and total antioxidant capacity exhibited a significant dose dependent decrease. The present study thus concluded that ROS mediated cytotoxicity and genotoxicity was exhibited by these metallic nanoparticles and hence its concentration at the disposal site of industrial effluents should be monitored. The study established WAG cell line to be used as model system for risk assessment of nanoparticles on aquatic animal health.
